# Cross-Participant EEG-Based Assessment of Cognitive Workload Using Multi-Path Convolutional Recurrent Neural Networks

**DOI:** 10.3390/s18051339

**Published:** 2018-04-26

**Authors:** Ryan Hefron, Brett Borghetti, Christine Schubert Kabban, James Christensen, Justin Estepp

**Affiliations:** 1 Department of Electrical & Computer Engineering, Air Force Institute of Technology, WPAFB, Dayton, OH 45433, USA; ryan.hefron@afit.edu; 2 Department of Mathematics & Statistics, Air Force Institute of Technology, WPAFB, Dayton, OH 45433, USA; christine.schubert@afit.edu; 3 Air Force Research Laboratory, WPAFB, Dayton, OH 45433, USA; james.christensen.7@us.af.mil (J.C.); justin.estepp@us.af.mil (J.E.)

**Keywords:** convolutional, recurrent, neural network, cognitive workload, MATB, EEG, cross-participant, mental workload, temporal specificity, ensemble

## Abstract

Applying deep learning methods to electroencephalograph (EEG) data for cognitive state assessment has yielded improvements over previous modeling methods. However, research focused on cross-participant cognitive workload modeling using these techniques is underrepresented. We study the problem of cross-participant state estimation in a non-stimulus-locked task environment, where a trained model is used to make workload estimates on a new participant who is not represented in the training set. Using experimental data from the Multi-Attribute Task Battery (MATB) environment, a variety of deep neural network models are evaluated in the trade-space of computational efficiency, model accuracy, variance and temporal specificity yielding three important contributions: (1) The performance of ensembles of individually-trained models is statistically indistinguishable from group-trained methods at most sequence lengths. These ensembles can be trained for a fraction of the computational cost compared to group-trained methods and enable simpler model updates. (2) While increasing temporal sequence length improves mean accuracy, it is not sufficient to overcome distributional dissimilarities between individuals’ EEG data, as it results in statistically significant increases in cross-participant variance. (3) Compared to all other networks evaluated, a novel convolutional-recurrent model using multi-path subnetworks and bi-directional, residual recurrent layers resulted in statistically significant increases in predictive accuracy and decreases in cross-participant variance.

## 1. Introduction

One critical area for research aimed at improving overall performance in human machine teams has been the development of models which better predict human cognitive workload: when a machine knows the human’s workload it can make better decisions. Many of these efforts use neurophysiological signals, such as electroencephalographic (EEG) data, to infer the cognitive workload that the human is experiencing while performing a task. As participants complete tasks their EEG signals are recorded and their cognitive workload is assessed by subjective ratings. Then the neurological signals and the workload measurements are used to fit a machine learning model which can infer the participant’s workload from the signals alone.

A historical standard for human model performance is the tailored single-participant model. Single-participant models are fit using only data from the participant being modeled, not data from other people. Since single participant models are specifically trained to perform well on the individual, these models will often have the highest performance with respect to that particular individual. However, training a separate model on each individual is resource intensive for both collection and processing. If the data for models could be collected from many people instead of just the one being modeled, the collection burden could be spread over many individuals.

Models trained on data from multiple people are known as group models or cross-participant models. In cross-participant model-fitting, data from one set of people is used for training and the models are later used to make workload predictions on those people, or possibly other people. The benefit of these cross-participant models is that they can be prepackaged and used in many settings with many individuals, requiring little or no calibration for each individual.

A model should perform well on an arbitrary individual independently of which set of other people it was trained on. However, individual differences apply here: variation in EEG response to workload between people makes it challenging to design, train, and validate models using data from some people to make good workload assessments on others. As in most other machine learning settings, a desirable model is one in which the predictions are accurate and the variance over predictions is low. Another desirable characteristic is being able to make accurate, low variance predictions on short EEG data sequences, as this should result in minimum lag in accurate assessment. However, machine learning models almost always make better predictions on longer streams of data, so there is a trade-off between temporal specificity and model performance.

In this paper, we explore the trade-space of model accuracy, variance, temporal specificity, and computational efficiency in three thrusts: (1) develop new tailored architectures designed specifically for cross-participant classification of EEG signals; (2) evaluate efficiency and performance of various training methods for these architectures; (3) characterize the effect of varying the temporal length of EEG features available to a model.

We present new methods for cross-participant estimation of operator workload in a non-stimulus-locked, multi-task environment by developing and evaluating seven neural network architectures. We specifically examine the case where no data from the test participant is used in any way to improve feature distribution similarity with the training or validation participants. Our findings show that a novel Multi-Path Convolutional Recurrent Neural Network (MPCRNN), designed to learn cross-participant frequency and temporal representations, simultaneously resulted in a statistically significant improvement in accuracy and decrease in variance compared to all six other highly-tuned network architectures using a MATB dataset with eight participants. This contribution moves away from reliance on the clinical frequency bands and towards learning of appropriate frequency representations—a significant departure from previous deep learning work in the field.

We show that increasing sequence length—a common method to improve accuracy in non-stimulus-locked settings—increases both mean accuracy and variance at statistically significant levels for cross-participant models. Since variance grows with sequence length, increasing temporal sequence length does not adequately address the cross-participant modeling challenge: Producing high-accuracy models with low variance across participants.

The remaining research thrust examines differences in performance, computational efficiency, and use cases of four training methods including single models incorporating all training participants and ensembles of individual-participant models. No significant differences were found between ensemble and group-based methods, so selection should be driven primarily by experimental design and computational cost constraints which generally favor the use of an ensemble. In addition to the primary thrusts, this paper fills gaps in current research by performing a direct comparison between different convolutional and recurrent network architectures in a multi-task, non-stimulus-locked environment. Multi-path convolutions and residual connections in bi-directional recurrent networks are both shown to have a positive effect on network performance over baseline models.

### 1.1. Related Work

Cross-participant modeling techniques use data from multiple individuals to fit statistical machine learning models that later are used to make predictions on people. These models can be divided into two categories: shared-data and zero-data methods. Shared-data methods fit models using data from all people including those on which the model will make predictions. Zero-data models use no data from the evaluated individual; these models only use data from other participants to fit the model.

In the first subsection, shared-data studies are reviewed. This subsection also examines cross-participant feature saliency. In shared-data cross-participant studies, finding data features which have good predictive value and are generalizable across all participants (salient features) is one of the goals. In the second subsection, zero-data studies are reviewed—these studies maintain a strict data boundary between the individual to assess and the set of individuals used to fit the model.

Application domains reviewed in this section include both cognitive modeling and medical prediction, which have similar desirable characteristics. Cognitive modeling applications cover detecting cognitive load, fatigue, attentional lapses and neural oscillations from movements or imagined movements. Medical field applications include epileptic seizure detection and classification. This section differentiates the use of time-locked stimulus models from non-time-locked models in the research. In a laboratory experiment, isolated, well defined, causal stimuli can be generated, allowing these stimuli to be used in time-locked models. In real-world multi-task environments, cognitive activity is not always associated with individual, causal, well-defined event stimuli. Because real-world tasks lack these well-defined stimuli, time-locked models may not be applicable.

#### 1.1.1. Shared-Data Cross-Participant Modeling and Feature Saliency

Several researchers developed group-trained models where a portion of data was used from each participant for training while the remaining data from those same participants were used for testing. Since these shared-data models were trained using some of the data for the individual being assessed, these models yield the highest possible expected classification accuracy for cross-participant models. Wang et al. [1] achieved 80% accuracy while showing a hierarchical Bayes model outperformed baseline neural network models in a three class, cross-participant MATB workload classification setting. Cross-participant variation was accounted for by learning parameters of a hidden Gaussian representation using segments from all eight participants’ data and testing on other data segments from those same eight individuals [1]. Zhang et al. [2] used adaptive exponential smoothing to improve feature stationarity and adaptive bounded Support Vector Machines (SVMs) to improve cross-participant generalization by iteratively adding misclassified examples from the test set to the training set to adapt the model performance to a new participant. Yin et al. [3] trained models using the transfer recursive feature elimination technique with linear SVMs and found that by adapting features from other individuals based on a small validation set from the test individual, statistically significant improvements in classification performance resulted when evaluating the remaining test data.

Wilson and Russell [4] conducted a within-participant MATB study and determined the relative contribution of different features from each individual varied widely among participants. This highlights the challenge of cross-participant distributional differences in the MATB environment. A similar result was reported by Noel et al. [5] for an in-flight workload experiment where a drastic difference in the number of salient features between pilots was noted. Additionally, for each pilot, most salient features differed across days causing salient features from multi-day experiments to diverge from single day experiments [5]. This indicates a coupling of temporal non-stationarity and cross-participant differences can compound the problem. In a separate MATB experiment, Laine et al. [6] also found feature saliency at the individual level to be highly variable, but were able to identify a stand-alone set of features that worked for training ArtificialNeuralNetworks (ANNs) on all individuals by using Stepwise Discriminant Analysis (SWDA) to select common features across the group. This yielded a binary classification accuracy of 83% which did not significantly differ from the within-participant modeling result. Their finding is important because it suggests when using all participants for feature selection, a set exists that does not reduce classification accuracy from the accuracy level achievable in individually-tailored models.

#### 1.1.2. Zero-Data Cross-Participant Modeling

Since the objective of many EEG application domains is to be able to deploy the technology with little to no user-specific data available for model tuning, numerous researchers have explored zero-data cross-participant modeling. For zero-data cross-participant models, this section examines how training methods, algorithmic assumptions, and features affect assessment accuracy and variance across participants.

While shared-data methods have shown that cross-participant model performance can approach within-participant accuracy in some cases, zero-data models often perform worse than within-participant models, due to individual differences. Gevins et al. [7] trained cross-participant single-hidden-layer ANN models using all individuals except the hold-out test participant for binary classification of stimulus-aligned spatial and verbal working memory tasks. The mean classification accuracy for the group classifier was 83% which represented a significant reduction from the 94% accuracy reported for individually-trained models [7].

In zero-data cross-participant modeling, different algorithms can significantly affect performance. Using Improved Performance Research Integration Tool (IMPRINT) workload profiles [8] as regression targets for a simulated remotely piloted aircraft tracking task, Smith et al. [9] showed algorithm type could have a statistically significant effect on zero-data cross-participant operator workload estimation for non-stimulus aligned tasks, a result confirmed by our work. Additionally, random forests improved group-trained model performance when compared to non-ensemble methods in the same algorithmic family, suggesting that in complex workload environments, ensemble models may yield better performance than their non-ensemble counterparts.

Now we discuss the impact of deep learning on zero-data cross-participant modeling. Certain deep neural networks outperform other methods for zero-data cross-participant modeling because they better model two of the conditions present in human state assessment: (1) the temporal ordering of signals which result from brain activity, and how those time-series signals map to temporally ordered sequences of human state assessment; and (2) the spatial relationship between EEG collection sites on the scalp. Temporal context can be accounted for using Recurrent Neural Networks (RNNs) and/or Convolutional Neural Networks (CNNs) while the spatial contribution can be modeled by CNNs.

Accounting for temporal context using Elman RNNs [10] improved diagnosis of epilepsy using cross-participant modeling of EEG data [11,12]. Utilizing cross-validated, group-trained models, Güler et al. [11] and Übeyli [12] reported reductions in diagnostic error of 63% and 74%, respectively, compared to non-recurrent methods. Recurrent networks were also effective in a high-fidelity vehicle simulator study using EEG to sense occipital lobe activity prior to and during lane perturbation events when performing a simulated highway driving task [13]. While the reported results indicated slightly better performance for ensembles of group-trained Recurrent Self-Evolving Fuzzy Neural Networks (RSEFNNs) compared to a battery of other neural network ensembles in predicting a normalized drowsiness metric [13], a rigorous statistical treatment in their study could have confirmed this. Despite the lack of statistical results, Liu et al. [13] demonstrated that ensembles of recurrent networks can produce excellent results in a stimulus-aligned cross-participant task environment.

Several researchers have accounted for both temporal and spatial relationships in EEG data by using CNNs or combinations of CNNs and RNNs. Lawhern et al. [14] developed a small CNN architecture that was able to generalize well across several EEG Brain Computer Interface (BCI) analysis domains including visual stimulation of P300 Event-Related Potentials (ERPs), neural oscillations associated with movement-related cortical potentials, and sensorimotor rhythms evoked by real or imagined movements [14]. Convolutions across the electrode channel dimension as well as temporal dimensions were used. The first layer of their model used 16 1-d kernels (each the same length as the number of electrode channels), which were convolved without zero-padding with each of the input tensors. This layer was the most interesting development of Lawhern’s model in that each of the kernels had the ability to learn useful channel interactions and had the effect of abstracting away the need to explicitly model locational dependencies inherent in an EEG system. Whenever possible within the constraints of a given dataset, Lawhern et al. [14] trained models using a cross-validated, cross-participant group method so that it was user-agnostic [14]. Importantly, Lawhern et al. [14] found that cross-participant variability of classification accuracy correlated with the Signal to Noise Ratio (SNR) of the signal associated with the phenomenon of interest. This means that for operator workload experiments in a non-stimulus-aligned environment such as the MATB, high cross-participant variability could be expected.

Hajinoroozi et al. [15] constructed two unique CNNs which were designed to perform convolution across 1 s temporal periods of raw EEG data from each channel resulting in improved cross-subject and within-subject classification for a driving simulator lane perturbation task compared to a large array of baseline algorithms. The CNNs convolved across the time domain in a manner which effectively searched for ERPs present in each individual channel. The first CNN used 10 kernels while the second used only one kernel, but was pre-trained as a Restricted Boltzmann Machine (RBM) followed by fine-tuning. The first CNN significantly outperformed all other models for within-participant prediction with an Area Under Curve (AUC) of 0.8608, while the RBM CNN performed far better than any other model in the cross-participant classification environment, achieving an AUC of 0.7672 [15]. These results suggest that either the reduced model capacity of the RBM CNN led to better cross-participant generalization, or that the process of performing unsupervised pre-training helped learn shared features across individuals. Overall, the use of an architecture which uses raw EEG to find per-channel ERP signatures was novel and warrants further investigation as a merged component in a large deep neural architecture which also incorporates time-frequency domain features.

Bashivan et al. [16] trained a deep convolutional-recurrent neural network to predict cognitive load during a working memory task [17]. A time-series of 3-channel images were created by performing 2-d Azimuthal Equidistant Projections (AEPs) of Power Spectral Density (PSD) features from the theta, alpha, and beta frequency bands. Models were trained using early stopping based on a randomly-selected validation sample selected from within the training set of a 13-fold, leave-one-participant-out train/test setup. Results showed a 30% reduction in error compared to random forest models and indicated strong frequency-band selectivity, meaning the filters applied to specific channels of input feature space [16]. However, since mean spectral powers in EEG clinical bands were used, and the definition of these bands were organically developed over a century of experiments, it is unlikely that features developed only from combinations of these bands will be optimal for all human state assessment activities. Models which can learn the most applicable frequency responses at a finer granularity may perform better and should be considered in future research.

In the recurrent models discussed so far, the temporal direction is always forward such that early signals influence the model’s understanding of later signals. This architecture ensures causality of brain activity is not violated but does not allow for reflection: a model cannot learn how to interpret the early signals using signals which are experienced later. An example of a type of signal in which reflection is important is speech. In the speech recognition task, an audio signal is converted into a string of characters or words. It is common to estimate the probability distribution of possible next words as conditioned on the signal and the previous words (or audio signal associated with those words). However, it is likely necessary that the conditional dependencies in speech be considered in both the forward and reverse directions to maximize transcription accuracy. Graves and Schmidhuber [18] showed that by using a model capable of understanding both forward and reverse dependencies in speech, performance was improved. The team used bi-directional Long Short-Term Memory (Long Short-Term Memory (LSTM)) units that could effectively exploit contextual dependencies in both directions to improve speech processing.

Recently, research using bi-directional LSTMs for brain signal analysis has begun [19]. Thodoroff et al. [19], implemented a bi-directional LSTM following a 2-d convolutional architecture and prior to a fully-connected layer for cross-participant epileptic seizure classification. Their architecture performed spatial convolutions similar to Bashivan et al. [16]. This combined with pooling layers enforced spatial invariance which is important for seizure classification since seizures can occur in any localized region of the brain, or globally [19]. Thodoroff’s reason for incorporating a bi-directional layer was because neurologists typically use both past and future information to make a diagnostic decision on whether an EEG segment contained epileptic activity [19]. Thodoroff’s application domain resulted in a minor limitation which needs further investigation if this technique is to be applied in real-time workload classification, because the task for this study was to use all the data to classify seizures. When all the data is available, bi-directional models can be used with impunity. However, in a real-time classification task, the future information is not yet available. Therefore, care must be taken to have a model in which the bi-directionality updates respect the lack of future knowledge—updates can occur backwards from the present towards the beginning of the current temporal-data stream, and separately, forward from the beginning of the temporal-stream to the present.

Of all research discussed thus far, none have used an ensemble of participant-specific, individually-trained models despite excellent performance of ensembles in other domains where distributional differences are present. Fazli et al. [20] used existing BCI data from 45 individuals across 90 sessions to train an ensemble of classifiers to identify imagined right hand versus left hand movement. The goal in this stimulus-aligned experiment was to create an ensemble which could handle cross-participant distributional differences and classify new participants with no prior data from the new participants. After training on this set, a separate hold-out set with 29 individuals and 53 sessions was used to assess model performance against various baselines. Final ensemble weightings of individually-trained Linear Discriminant Analysis (LDA) models were determined using $\ell_{1}$ regularized quadratic regression to select and reduce the number of classifiers in the ensemble to relevant ones [20]. Cross-validation was used for model tuning [20]. Their results indicated that using ensembles of individually-trained classifiers can improve classification accuracy over traditional group-trained models (30.1% and 36.3% error respectively) and perform comparably to models trained and tested on the same individual (28.9% error) [20].

In summary, choice of model type and training methodology have been shown to have an effect on cross-participant EEG analysis across a variety of applications. Ensemble methods, CNNs, and RNNs have generally improved results. However, no comparison using different training techniques has been characterized for deep neural network models. Additionally, aside from medical applications, cross-participant research using deep architectures used some form of stimulus to time-align the signals for analysis. A drawback to stimulus-aligned models is that most human tasks in real world environments do not experience time-locked stimuli; instead, humans often work in multi-task environments and make arbitrary decisions when to switch attention or tasks—exemplified by the MATB environment. In these unconstrained environments, obtaining temporal-specificity on environment changes or task switches is difficult, and we estimate that models that require stimulus-aligned information will have difficulty performing well in such environments. Since deep neural network techniques have not yet been applied to non-stimulus-aligned task environments such as the MATB for cross-participant analysis, performance in these environments is unknown. Furthermore, performance of ensemble-of-individual-participant models has not been characterized using deep neural networks despite their effectiveness as described by Fazli et al. [20]. Finally, while shared-data modeling methods are commonly used in research when the number of participants in a study is low, ultimately the field should move toward zero-data methods because they do not require model refitting each time predictions are to be made on a new individual.

In the next section we describe additional advances in deep learning architectures which will be fruitful in addressing some of the shortcomings of existing research.

### 1.2. Applicable Advances from Computer Vision (Multi-Path Modules and ResNets)

Two of the primary advances in recent years that have pushed the computer vision field to new heights of accuracy are multi-path subnetworks and deep residual networks (ResNets). These advances went mainstream in the different variants of Szegedy’s GoogLeNet architecture, which use multi-path subnetworks, and the development of the numerous instantiations of ResNet [21,22,23,24]. The main idea behind GoogLeNet is the *inception* module. Each *inception* module considers what type of local structure is required for a vision task since it can then be repeated spatially [22]. The most important contribution associated with the inception module was the idea of enabling multi-scale processing at a local level. To do so, each inception module takes the output tensor from the previous layer and passes it through several different-sized convolutional layers as well as one max-pooling layer in parallel, with appropriate padding to ensure consistent output dimensions [22]. The output from each of these operations are like-sized feature maps with varying depths that can be concatenated in the depth dimension. This results in a diverse set of features being learned at each layer.

He et al. [23] developed ResNets to address optimization problems associated with training very deep networks. The main idea behind a ResNet is to introduce unparameterized identity skip connections that change the layer-wise learning problem to one of learning the residuals rather than trying to learn an unreferenced output. Residual learning can be mathematically described as:

(1) $$y=F(x,W_{i})+x,$$

where *y* is the output vector, *x* the input vector from the unparameterized identity skip connection (added to force a residual mapping), and *F* is the function learned by the intervening layers as a non-linear function of the input vector and weights, $W_{i}$ [23]. He et al. [23] compared training/validation performance of 18 and 34 layer networks each of which had two incarnations: a plain network (no skip connections) and a version whose only difference was the addition of the skip connections every two layers to force a residual mapping. They found that their training error for a 34-layer network was higher than the training error of an 18-layer network for the non-ResNet networks and confirmed it was not due to vanishing gradients by checking the gradient norm at each layer [23]. This indicated that the optimizer was unable to find a solution as good as a simple 16-layer identity mapping (input layer, 16 identity mapping layers, output layer) which led them to believe the problem with training deep feed-forward convolutional neural networks was challenges with non-convex optimization [23]. Empirically, they discovered that using residual learning resulted in network optimization working well even for extremely deep networks. Furthermore, they determined that increasing network depth seemed to monotonically decrease classification accuracy up to networks of 1000 layers, showing that adding network depth improves representational learning [24].

He et al. [24] also proposed pre-activation: a new ordering to apply batch normalization, activation functions, and then convolutions. This ordering led to a dramatic 35% reduction in test error as network depth increased. An important consideration when using pre-activation in a network is how to handle the first and last activations. It is recommended to initially use a convolution directly followed by an activation for the first elements in the network [24]. Following the last element-wise addition in the network, an extra activation function is recommended [24]. Furthermore, it is important to note that dropout should not be used along the skip connection [24].

Szegedy, et al. expanded upon He’s work and applied residual networks to their GoogLeNet architecture [25]. They showed that scaling the residuals by a factor of between 0.1 and 0.3 improved stability during training and suggested this technique rather than making large adjustments to the learning rate for tuning the training. They also found that incorporating residual connections into their multi-path architecture caused training time to decrease drastically [25].

In summary, applicable advances from computer vision research include:

- Distribute computational load evenly across layers, while scaling network depth and feature map depth together with appropriate reductions in individual feature map size [21].
- Instead of using large convolution kernels, stack layers of smaller kernels to attain the same receptive field with significantly reduced parameters and computational requirements [21].
- Use batch normalization to produce stable input distributions prior to activation functions [24].
- Reduce computational load and overfitting, and improve interpretability by using global average pooling [21,26].
- Use full-length kernels in one dimension to identify correlations among distant regions depending upon the tensor’s structure [14].
- Scale residual activations by a factor of 0.1 to 0.3 prior to summing with the identity connection from the previous layer to improve training stability [25].

### 1.3. Applicable Advances from Natural Language Processing (LSTM and Bidirectional LSTM)

RNNs have been used in natural language processing due to their ability to account for temporal context in speech and text. However, vanilla RNNs have struggled to model longer contexts well due to the mathematical sensitivities during the training process. These problems are referred to as vanishing or exploding gradients, and they lead to poor performance of vanilla RNNs. An LSTM is a gated recurrent neural network that was designed to overcome the vanishing or exploding gradient problem [27]. The LSTM was developed by Hochreiter and Schmidhuber [28] and improved with the addition of a forget gate by Gers et al. [29]. A recent, detailed description of the LSTM is available in Hefron et al. [30].

One limitation of the LSTM is that it can only process the temporal stream of observations in the forward direction, making it inefficient for the network to learn how earlier elements in the stream might be conditionally dependent on later elements. Bidirectional LSTMs were developed to address this inefficiency. A Bidirectional LSTM (BDLSTM) can be conceptualized as two LSTMs with their outputs concatenated at each time step in the sequence. What makes it bidirectional is that one LSTM processes sequences in their original temporal ordering while the other processes sequences with the temporal order reversed. The advantage bi-directionality brings is that future context can inform past events. This even works for real-time environments, because the current state may be understood more clearly by processing older data in the context of newer information.

Bidirectional networks have proved useful in a variety of natural language processing tasks [31]. Because humans think in terms of language and plan actions with an understanding of temporal context, it is a reasonable expectation that brain activity may be better understood by considering its bidirectional context; bidirectional networks may perform well in human state assessment tasks.

## 2. Materials and Methods

Our research focuses on a sub-goal of human state assessment: estimating cognitive workload. To accomplish our exploration of new deep architectures which perform well in assessing cognitive workload, we first obtained a dataset. The remainder of the work can be divided into four efforts: data preprocessing, network architecture design, network training, and development of our performance evaluation strategy.

### 2.1. Dataset

The dataset was collected during a human research study focused on neural sensor fusion. Eight participants completed four blocks of tasks over the course of two sessions in a single day with two blocks being performed in each session. One participant was left handed and two of the eight participants were female. Participants varied in age between 19 and 27 with a mean of 21.9 and standard deviation of 2.57 years. Each block in this experiment consisted of five-minute long trials of high-workload MATB [32], low-workload MATB, as well as verbal N-back 0, N-back 1, N-back 2, and N-back 3 presented in different orders within each block to control for fatigue and/or practice. This resulted in 30 minutes of data per participant per block. This study only analyzed the MATB data.

All four MATB subtasks were performed during both the low and high workload conditions. Temporal density of stimuli was adjusted to ensure a difference in task difficulty and performance between the two conditions. The low difficulty task setting was the same for all participants while high difficulty settings were individually tuned. Four 2-factor ANOVAs were used to determine if there were differences in mean performance metrics between low and high workload MATB tasks blocked by participant and with allowance for a participant by workload interaction. The two factors were workload with 2 levels (low and high), and participant with 8 levels: one for each participant. Each ANOVA corresponds to a subtask performance metric gathered during all trials in the experiment. The tracking task metric represents the percentage of time the aircraft flight path vector was in the desired region. The system monitoring task metric is the percentage of abnormal lights and dials conditions for which the user made the appropriate correction prior to the condition timing out. The communications metric tracks percentage of correct responses to auditory inputs. The resource management task metric represents the proportion of time both tanks were within operational limits.

Table 1 shows that statistically significant differences in mean performance existed between low and high workload conditions for the tracking and system monitoring tasks for all participants. Additionally, significant differences in low versus high workload mean performance for the resource management task were present for only participants 3 and 7.

Participants were trained to asymptotic performance in the tasks during five training days prior to the test day to ensure learning effects were minimized during collection of the experimental data. The ordering of the scenarios varied by participant and by session in a randomized block design to control for ordering effects. Five-minute long baseline trials were performed at the beginning of each block and a 30-min break separated the second and third blocks. Baseline segments were not used in our analysis. For each of the participants in the study, 128 EEG channels conforming to the BioSemi equiradial layout, along with 2 mastoid electrodes, 2 Horizontal Electrooculograph (HEOG) channels, 2 Vertical Electrooculograph (VEOG) channels, and 2 electrocardiogram (ECG) channels were recorded at a sampling rate of 4096 Hz using the BioSemi ActiveTwo recording system (BioSemi B.V., Amsterdam, The Netherlands). The skullcap was never removed during the test day and electrode channels were monitored throughout each session to ensure the highest data quality. Although not used in our analysis, 38 functional Near-Infrared Spectroscopy (fNIRS) channels were simultaneously recorded during the experiment and were interspersed with the EEG electrodes. Analysis was performed both including and excluding the single left handed individual due to possible motor cortex and prefrontal cortex differences. Since no significant changes in results were present based on inclusion or exclusion of this individual in analysis of the MATB experiment, the left handed participant’s data was used for all analysis in this study.

### 2.2. Data Preprocessing

A substantial amount of preprocessing was required. It largely focused on cleaning the data, downsampling both spatially and temporally to reduce input feature space, calculating PSD, performing normalization, and populating input-tensors of various shapes to match the desired structure for each of the architectures being explored.

All participants’ data were trimmed to 303 s per trial, downsampled to 512 Hz and down-selected to 64 electrode channels (approximating the extended EEG 10/20 montage) to reduce computational complexity. The PREP pipeline [33] was used to identify and interpolate bad channels, calculate a robust average reference, and remove line noise. A high-pass filter with a cutoff at 1 Hz was then applied to the data. PSD at frequencies between 3 and 55 Hz inclusive were computed for each electrode channel using a 2-s Hanning-windowed Short-Time Fourier Transform (STFT) with an overlap of 1 s. This resulted in 3392 features for every 1-s observation.

Since the eventual goal is to use this type of system in real-world, real-time environments, it is undesirable to remove data segments containing EEG artifacts because their removal would lead to breaks in prediction. Rather, our aim was to appropriately mitigate the effects of numerous high-variance artifactual segments that had potential to cause poor performance by identifying these sections and modeling through them. Since the purpose of this research was not to demonstrate a new artifact detection technique, we manually marked high-variance segments with the assumption that a real-time tool would be capable of marking similar segments. The high-variance segments were then temporarily ignored during normalization so that these sections would not negatively impact the normalization procedure. Due to the convolutional nature of the STFT, it was important to also ignore a window of ±1 s on either side of an artifactual segment in order to capture all points that were affected by a high-variance artifact during computation of PSD values.

To normalize the PSD values, an empirical Cumulative Distribution Function (CDF) was created for these values at each time step for each electrode/frequency combination within a given participant’s block for the MATB task. An empirical CDF is a non-parametric normalization method which maps values ordered from lowest to highest into corresponding percentiles based on position. In our case, we used the computed CDF values to transform the PSD values and scaled the results to span the range [−1,1] rather than [0,1], as this was advantageous for neural network training.

Next, the dataset was repopulated with the previously-ignored high-variance artifact segments. Several possibilities were examined regarding reinsertion of these segments into the already normalized data. Empirically it was determined that best algorithmic performance could be achieved by replacing these high-variance EEG feature segments with zeros, and, for all architectures except CNNs, the data stream was augmented by concatenating a boolean bad-data indicator variable which was set to 1 in these segments and zero elsewhere. Due to the way parameters are shared in a CNN, adding an indicator variable would have been detrimental, so this technique was not used for that architecture. An alternative normalization process would have been to apply the empirical CDF to all the data, including the high-variance segments. This was attempted, but these segments consistently produced saturated results of −1 or 1 which degraded overall system performance. By setting the high variance segments to 0 and the boolean indicator variable to 1, temporally stateful models are able to learn the best way to propagate the state through these segments.

**Figure 1:**
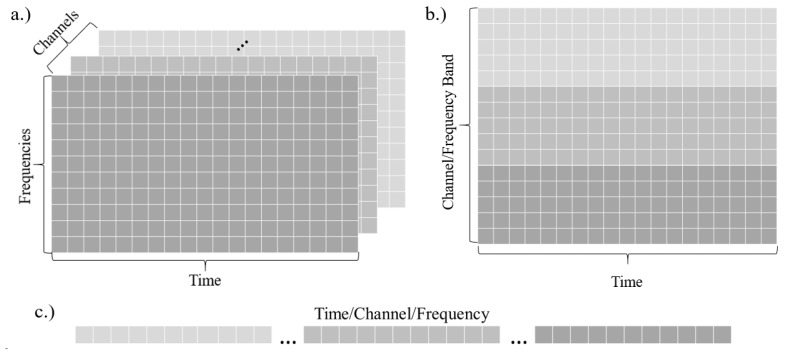
Input shapes for each network type are illustrated: (**a**) The CNN input shape was expanded about the channel (electrode) dimension as the number of filters increased; (**b**) The LSTM input flattened the electrode and frequency dimensions into a single combined dimension; (**c**) All dimensions were flattened into a single input vector for the ANN.

Finally, rolling 5, 10, 20, and 30-s windows with a step size of one second between windows (4, 9, 19, and 29 s of overlap respectively) were created to prepare features for input to convolutional or recurrent networks. For the feed-forward ANN, all features in the sequence were flattened and provided as a single vector to the network. Figure 1 depicts the different input shapes to the various types of networks.

### 2.3. Model Architectures

Numerous deep neural network architectures were built to evaluate their effectiveness in a multi-task environment. Here, we briefly describe the design of seven neural network architectures. Detailed depictions of each architecture are available in the Supplemental Materials. Several architectures are derivatives of others, so we begin by describing baselines and building up in complexity. To avoid repetitiveness, all network specifications only describe the hidden layers and will forgo detailing the input layer shapes and sigmoid output layers used for workload classification.

Two neural network architectures were used to provide neural network performance baselines for the more complex architectures. A single-hidden-layer ANN was trained while varying the number of hidden nodes to examine validation performance for models containing between 1 and 6 million parameters for each sequence length. On average, models with 64 nodes performed the best on the validation data across sequence lengths. Hence, 64-node models were chosen as the final architecture. Appropriately-tuned $\ell_{1}$ regularization ($\ell_{1}$ rate =0.00000125) proved to be very important due to the large number of features in the input vector–101,790 features for the 30 s sequence. A two-layer LSTM (2L-LSTM) was used as a second baseline. This architecture was used in a previous within-subject MATB study [30]. The hidden layers consisted of a sequence-to-sequence LSTM layer with 50 memory cells followed by a many-to-one LSTM layer with 10 memory cells.

The convolutional architecture (CNN) consisted of a series of 3×3 kernels with Rectified Linear Unit (ReLU) pre-activations, 2×2 max-pooling layers, dropout, and batch normalization layers. The architecture convolved and pooled across time and frequency dimensions in an attempt to have the CNN learn meaningful temporal and frequential representations of PSD. Following the final convolutional layer, a 2d global average pooling layer was used to reduce computational complexity by reducing the overall number of parameters prior to a fully-connected layer. This layer fed into a fully connected layer which found correlations between average time-frequency feature maps and sent them to the output layer.

The remaining architectures are all either variants of a five-layer LSTM or larger models which incorporate a five-layer LSTM into the overall architecture. Several variants of each of these architectures were evaluated based on validation performance including 50, 110, and 200 unit-per-layer LSTMs. The 110-unit-per-layer variant was selected and trained for all final models. All LSTM models used 20% dropout on the input sequence. The first five-layer LSTM model was simply connected to the output layer with no further modification. The second variant, a BDLSTM, examined the effect of bi-directionality on the model by making each layer a bi-directional LSTM without any other architectural modifications. The final variant, the Bidirectional ResNet LSTM (BDRLSTM), added residual connections around the middle three layers using an activation scaling factor of 0.3 to improve stability during training, as recommended by Szegedy et al. [25]. While Szegedy et al. [25] found that incorporating residual connections into their convolutional architecture caused training time to decrease drastically, we found a slightly increased number of training epochs were required to reach maximum accuracy using the BDRLSTM compared to the BDLSTM.

We call the final architecture a MPCRNN. This is the most complex design of the seven. It combines a wide multi-path, residual, convolutional network with a bi-directional, residual LSTM. The architecture finds multi-scale time-frequency relationships by simultaneously convolving or pooling PSD values across a variety of convolution and pooling paths while maintaining temporal and frequential structure. The notion of electrode channel is abstracted as the depth of the network increases.

A feature present throughout the convolutional sections of the MPCRNNs are 1×1 convolutions. Lin et al. [26] described how using 1×1 filters with a different depth than the preceding layer acts as a form of cross-channel parametric pooling. Szegedy et al. [22] used this idea to reduce the dimensionality of the inputs to the larger filters and to create compressed representations of the previous layer with the additional benefit of adding a nonlinearity. Our architecture borrows this idea in two ways. First, each multi-path module has a path that includes only 1×1 convolutions which means that all the diversity of scale from the previous module passes through to the next module in a compressed form. This multi-scale processing allows for unique and differently structured time-frequency representations to be learned. Secondly, each module uses 1×1 convolutions to reduce dimensionality and evenly distribute the computational load among the various paths.

**Figure 2:**
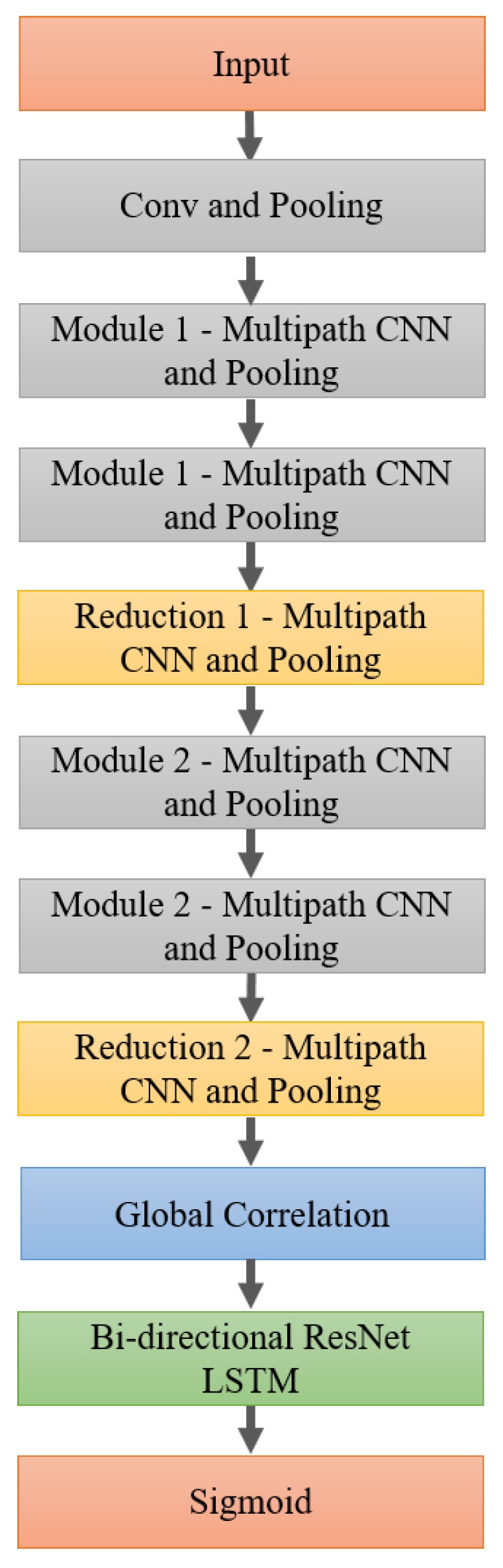
A modular depiction of the MPCRNN.

Figure 2 displays a modular view of the architecture, and a full schematic is presented in the Supplementary Materials. The overall model begins with a 2d convolutional layer in time and frequency followed by a max-pooling layer which downsamples the frequency space by a factor of 2. This representation is then fed into the first multi-path convolutional module, Module 1, which is repeated once with each instance employing residual connections to improve gradient propagation through the deep architecture. Module 1 begins with batch normalization, ReLU activation, and 20% dropout layers and then branches into five separate paths. Three paths have convolutional elements while the other two are pooling paths. All paths with convolutions utilize 1×1 convolutions to reduce the dimensionality of the inputs prior to the larger filters and to create compressed representations of the previous layer with the added benefit of adding a nonlinearity [22,26]. The purpose of the multi-path structure is to place no undue preconditions on the way frequency or time are represented. The convolutional and pooling model only enforces locality of time and frequency, but lets the network learn the appropriate scales of locality to make the features the most discriminatory. Max-pooling and average-pooling were included as separate paths because for some frequency bands, the maximum PSD may be the best feature, while in others, the average PSD over the entire band may be more useful. Each module also contains a path which includes only 1×1 convolutions which means that all the diversity of scale from the previous module passes through to the next module in a compressed form. At the end of each module, the various paths are concatenated, adjusted to the appropriate dimensionality via a 1×1 convolutional layer, and go through an activation scaling layer [25] prior to being added to the residually-connected features. Module 2 is very similar to Module 1 except that it handles different shapes since it is further downstream.

After each set of modules comes a multi-path frequency reduction module to downsample in frequency and expand in the channel dimension. After the series of multi-path and reduction modules, a 1×1 convolutional layer is used to reduce dimensionality prior to passing through a convolutional layer just prior to the BDRLSTM. This layer is unique because the kernel is the full width of the feature vector similar to the first layer of the architecture proposed by Lawhern et al. [14]. This finds global correlations across combinations of abstracted-electrode/convolved-frequency-band representations for each time period. After a reshaping of the tensor, the features are ready to be processed by the same BDRLSTM to account for temporal context, as previously described. An additional benefit of using residual connections in this architecture is that they allow gradients to propagate via identity connections to any residually-connected layer in the network, thus bypassing many layers when needed. We postulate that this is important for architectures which connect large recurrent networks on the end of convolutional architectures as it creates more direct paths to lower layers, thus improving the learning process.

### 2.4. Neural Network Training

After preprocessing was completed, the seven architectures were initialized, trained, and tested using a variety of techniques. Several zero-data cross-participant training methods were compared: a cross-validated group method, a validation-set group method, and two variants of cross-validated ensemble methods. Four sequence lengths ranging from 5 to 30 s of PSD features were evaluated for each model to understand the effect of sequence length and network architecture design on zero-data cross-participant workload classification.

While several variations in model-training were used in the course of this study, numerous network training considerations remained the same. We begin by describing the model development procedure and training aspects that were consistent across techniques.

All neural networks were trained using the Keras [34] and Tensorflow [35] frameworks. Model development was initially conducted manually using a patience-based early-stopping with validation set approach to tune model parameters such as regularization rate, number of hidden nodes, dropout rate, and architecture selection. This was to reduce model development time. Upon selection of final architectures, optimal-stopping methods were used to determine the number of training epochs based on validation data. Each network was trained using mini-batch gradient descent with a batch size of 128 observations. The Adam optimizer was used due to its ability to handle non-stationary targets and noisy data [36]. Learning rate values ranged from 0.0001 to 0.000001, depending on the model being trained, to ensure smooth training. All models used a binary cross-entropy loss function. Several forms of regularization were applied to each model including dropout [37] and various levels of $\ell_{1}$ or $\ell_{2}$ regularization. Next, the four cross-participant training methods are presented. These training techniques were used for all network architectures and data sequence lengths.

#### 2.4.1. Cross-Validated Group Method

Cross-validated training of each group model began by iteratively sequestering one participant’s data as the hold out test set. Next, 7-fold cross-validation was performed using each of the remaining seven participants’ data to tune number of training epochs. During cross-validation, models for each fold were trained for 30 epochs and were saved at each epoch during training. Validation accuracy was then averaged across folds at each epoch. The epoch with the highest average validation accuracy was selected as the best stopping epoch. After determining the best number of epochs to train based on cross-validation, the selected seven participants’ data was used to train a final group model to evaluate the hold-out test set (the 8th participant). This procedure was repeated until each participant had been used as the hold-out test participant once.

#### 2.4.2. Validation-Set Group Method

**Figure 3:**
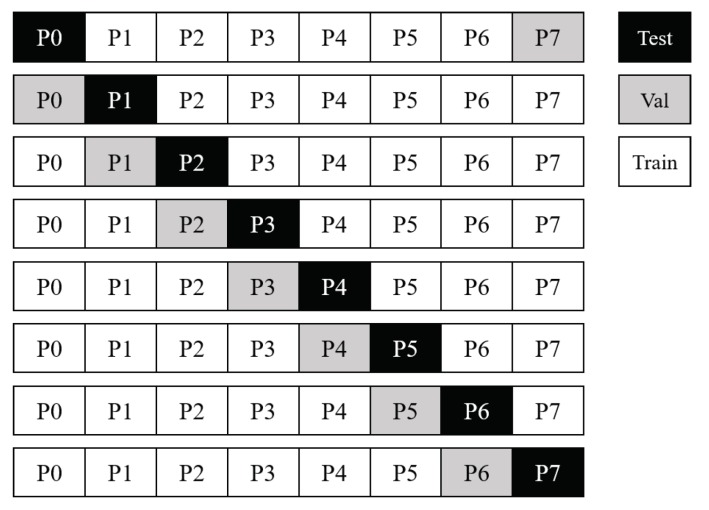
Training, validation, and test sets for the optimal-stopping validation-set group method are colored by use for each participant. For example, the fourth row indicates the model was trained using data from participants [0, 1, 4, 5, 6, 7], validated using participant 2’s data, and evaluated using participant 3 as the hold-out test set.

Architectures were also trained using the optimal-stopping group method as depicted in Figure 3. Models were trained using six participants’ data, validated using a seventh, and tested on an eighth participant in a leave-one-participant-out manner. In each case, the model was trained for 30 epochs with the model being saved any time better validation performance was achieved. This optimal-stopping method differs from a patience-based approach often used to train deep networks in that the selected training epoch is the one in which the network’s performance on the validation set was the highest over all epochs. This method contrasts with commonly-used patience-based early stopping, where an epoch counter is reset when a better performance is achieved. In the patience-based method, training continues as long as the epoch counter fails to exceed a pre-determined value. If a new minimum validation error is not achieved within that epoch count, training is stopped and the model associated with the best prior validation set performance is selected to train the final model. This final model is then used to make predictions on the associated hold-out-test participant’s data.

Using optimal stopping instead of patience-based stopping can never result in a worse validation-set accuracy. The optimal stopping method often yields a slightly better performing model than the patience-based approach, at the expense of an increase in training time. However, this additional computational expenditure was necessary to allow direct performance comparison with the cross-validated method which also used the optimally performing epoch found during cross-validation. All other network hyperparameters were consistent with those in the cross-validated group method.

#### 2.4.3. 7-Classifier Ensemble-of-Individuals Method

**Figure 4:**
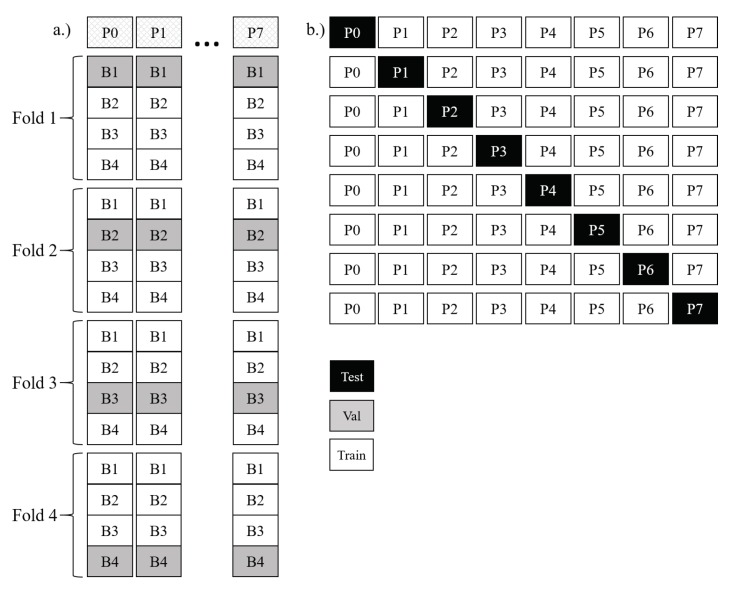
Ensemble Training: (**a**) 4-fold cross-validation across blocks was performed to tune hyperparameters for each within-participant model. Each of the models from these folds was also used as members of an ensemble of by-participant ensembles rather than continuing with part b; (**b**) Next, using each participant separately as the hold-out test set, the remaining individuals were used to train individual models using the hyperparameters derived from their individual model tuning in part a. Participants are not connected as they are in Figure 3 to highlight that they are each trained individually.

A simple ensemble of single-participant models served as the 7-classifier ensemble method. Individual model development began by splitting each participant’s data into the four blocks described in Section 2.1. Next, the number of epochs to train each of the seven architectures, outlined in Section 2.3, was tuned for each participant using 4-fold cross-validation. Final individual models were then trained using all data for each participant. A maximum of 240 epochs was used in training each model to ensure an equivalent number of backward passes were available to fit the network as in the group method. A visual depiction of this process is shown in Figure 4a,b respectively. The result was eight final models (one per participant) for each network architecture.

Throughout the training and cross-validation process, only one participant’s data was available in each model. Because of this, any combination of participant models can be chosen to form an ensemble to make predictions on another participant not in the ensemble. An additional benefit of the ensemble method is that it is computationally more efficient than the cross-validated group method, requiring less than 2/3 of the computational resources and time as the cross-validated group method. By tuning the learning rate differently, a further reduction in resources by a factor of 8 could be realized without impacting classifier accuracy. While this demonstration was performed, we opted to maintain consistency between as many parameters as possible for the across-training-method evaluations and report on the results as described. Final predictions for the 7-classifier ensemble consisted of a simple average of all participant’s individual probabilities for each class, with the highest average probability being the predicted class.

#### 2.4.4. 28-Classifier Ensemble Method

A second ensemble was formed using each of the four models per participant illustrated in Figure 3a. Rather than using each set of four models to determine a cross-validated stopping point and training with all the data for an individual participant, the overall ensemble just includes each of the four models across seven participants for a total of 28 models in the ensemble. Since no tuning was accomplished using any other models, there are never interactions between the hold-out test set data (any single selected participant), and the 28 models associated with the remaining participants. Again, a simple average of class probabilities across the models in the ensemble was used to make predictions on the test participant. This was performed iteratively using each participant’s data as the test set once. Testing this model against the others also allows for conclusions to be drawn regarding whether using a cross-validated stopping technique or an ensemble of by-participant-ensembles is more effective for handling distributional diversity attributable to individual differences.

This method required less computational resources than any other cross-validated method, reducing the 7-classifier ensemble computational requirement by approximately 20%. A final benefit to using ensemble training methods is that they are easy to update and improve as data from new individuals are obtained. This is because a new model is simply created and appended to the ensemble whenever new data is gathered. Conversely with the group training method, the entire model using all collected data needs to be retrained every time new data is added. This aspect of ensembles is especially attractive for real-world settings or long-term studies.

### 2.5. Statistical Evaluation Strategy

Several ANOVAs with post-hoc Tukey Honest Significant Difference (HSD) tests were conducted to understand how network architecture, sequence length, and training method affect both mean classification accuracy and variance of cross-participant classification accuracy. We believe that considering cross-participant variance of classification accuracy is required to appropriately characterize the effect of cross-participant distributional differences rather than merely examining overall accuracy. If a particular methodology reduces this variance while simultaneously improving mean accuracy, it could suggest a path to reduce the impact of cross-participant differences in future experimental designs. Unless otherwise stated, the results of the baseline CNNs models were omitted during statistical analysis due to their poor outlying performance. Throughout the statistical testing, an α=0.05 was used as the threshold of significance.

Statistical evaluation began with two 3-factor ANOVAs used primarily to understand the effects of training methodology in the context of a given network architecture and sequence length. The independent variables in each ANOVA were: network architecture with six levels, sequence length with four levels, and training method with four levels. Main and first-order interaction effects were examined. A Tukey HSD test followed each ANOVA to identify which levels statistically differed and to determine direction and magnitude of the differences. A single outlying datapoint associated with LSTM model performance was omitted during the ANOVA and post-hoc tests in order to satisfy model assumptions. This datapoint is further discussed in Section 3.1.

Because there were significant interaction effects associated with some of the training methods, two follow-up 3-factor ANOVAs were conducted. These ANOVAs were used to examine the effects of sequence length and architecture in the context of the two recommended training methods: the cross-validated group model and the 7-classifier ensemble method. The independent variables in these ANOVAs were: network architecture with six levels, sequence length with four levels, and training method with two levels. While only main effects were examined for the mean-accuracy follow-up ANOVA, the interaction of sequence-length and training method was considered for the variance of cross-participant classification accuracy ANOVA. A Tukey HSD test followed each ANOVA to identify which levels statistically differed and to determine direction and magnitude of the differences.

## 3. Results and Discussion

**Figure 5:**
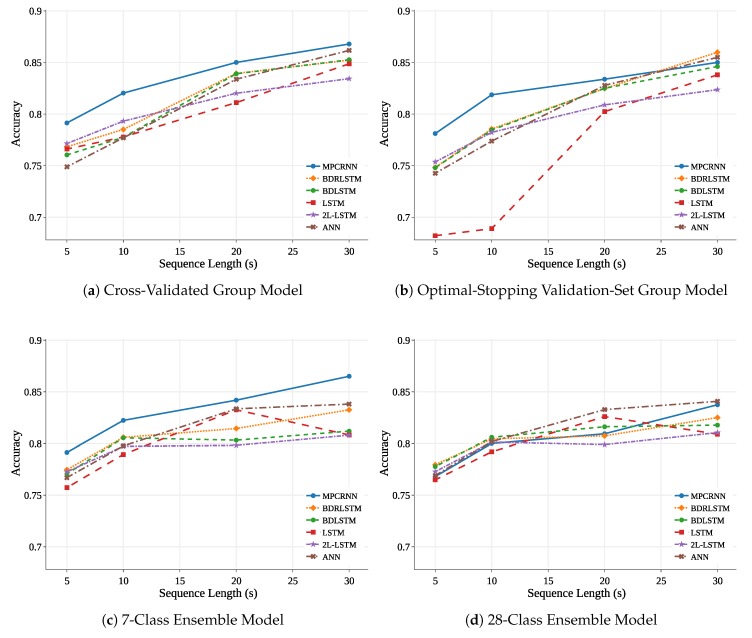
Mean classification accuracy for all participants as a function of sequence length, architecture, and training technique. Mean classification accuracy tends to improve as a function of sequence length for all classifiers and training methods. Error bars are omitted for clarity.

Now we decompose the various aspects of zero-data cross-participant models, dissect the key factors influencing model performance in order to characterize the domain, and make recommendations for future work. Overall results are shown in Table 2 and Figure 5. The following meaningful findings are discussed in detail in the ensuing sections and should inform future EEG-based analyses:

1. The cross-validated group training method and 7-class ensemble method performed similarly across shorter sequence lengths, but diverged at longer lengths due to a reduction in training observations associated with the ensemble method. Despite this, these methods are clearly preferable to the remaining methods and are suggested for future cross-participant EEG modeling depending upon application considerations, quantity of within-participant data, and computational constraints.
2. Increasing sequence length improves cross-participant mean accuracy, but also increases cross-participant variance. This indicates that a reduction in distributional dissimilarities between individuals cannot be achieved by increasing sequence length. To alleviate the problem of individual differences, a method which improves accuracy and decreases cross-participant variance is needed.
3. Compared to all other tested model architectures, the MPCRNNs architecture both improved cross-participant mean accuracy and decreased cross-participant variance. This demonstrates that using domain-specific knowledge to inform deep neural network architecture design can reduce the impact of individual differences on model performance.

We begin by explaining the effect of training methodology and then down-select to the two recommended training methods in order to more clearly understand the effects of sequence length and network architecture on model accuracy and cross-participant variance.

### 3.1. Effect of Training Method

Table 2 and Figure 5 depict cross-participant zero-data classification accuracy for each model as a function of PSD sequence length and model training method. The most prominent trend was associated with sequence length: as sequence length increases, accuracy improves. However, gains in accuracy associated with longer sequences were influenced by the chosen training method. Results of the mean classification accuracy ANOVA showed that the interaction of training method and sequence length had a significant effect on cross-participant mean accuracy (Table 3, p<0.0001). Table 4 highlights significant differences associated with the complex interaction between training method and sequence length. For 5 and 10 s sequence lengths, the validation set group model significantly underperformed all other training methods (all p≤0.0142). At a sequence length of 20 s, the cross-validated group model resulted in improved accuracy compared to the validation group model (p=0.0427) and the 28-classifier model (p=0.0090). Finally, at the 30 s sequence length, both group models outperformed the ensemble models (all p≤0.0392).

The complex interaction of training method and sequence length stems from the strengths and weaknesses of each training method. Ensemble models generally produced diminished gains in accuracy compared to the group models as sequence length increased. This effect is evident in the inferior performance for the ensemble methods at the 30 s sequence length as shown in Table 2. Compared to the group models, as sequence length increases, the amount of data used for validation of the ensembles consumes a larger fraction of the total training data available. This reduction becomes most critical when fitting complex models using the 28-classifier ensemble, which has only 2/3 of the training data available compared to the 7-classifier ensemble, and appreciably less data than the group models. The reduction in performance highlights an important consideration: determining the appropriate mixture of within-participant to cross-participant data to optimize predictive performance of the selected training method and architecture. If conducting an experiment with a large number of participants, we suggest gathering twice the data from the first few participants. This way, models can be fit to those participants’ data and it will be possible to examine the effect of varying the amount of training data available on a model’s performance prior to collecting data from the rest of the individuals. Then, an informed decision about whether to reduce data collection for the remainder of the participants can be made. Alternatively, this type of analysis can be completed using applicable preexisting data if available.

**Figure 6:**
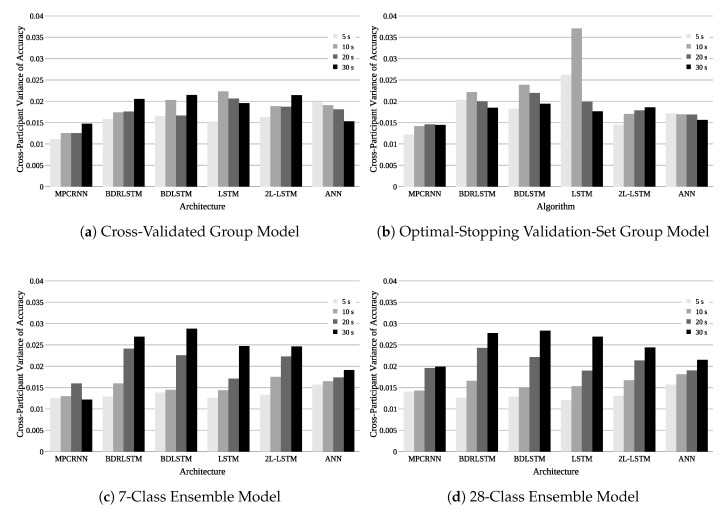
The plots of cross-participant variance of classification accuracy per architecture and sequence length show how much variance there was across-participants for a given training technique.

**Figure 7:**
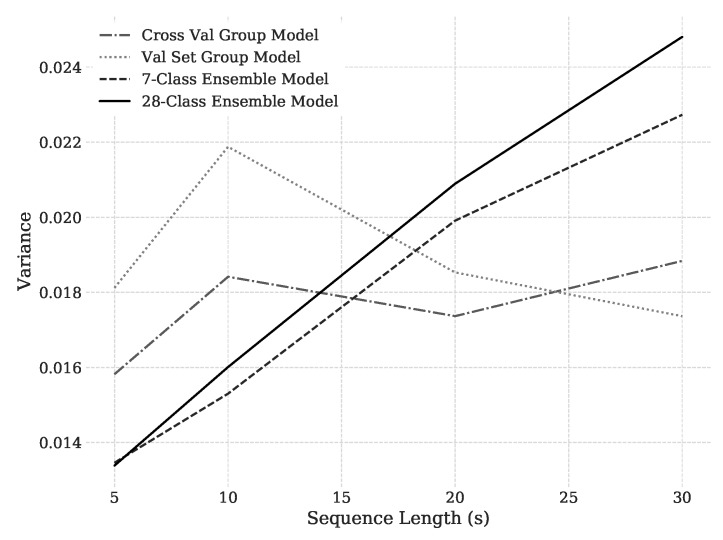
Interaction of sequence length and training method for cross-participant variance of accuracy.

A significant interaction between training method and sequence length was also present in the cross-participant variance of classification accuracy ANOVA (Table 5, p<0.0001). Visual inspection of Figure 6 corresponds with the ANOVA results from Table 5 and clearly shows the interaction effects. An interaction plot of training method and sequence length for variance is shown in Figure 7. It illustrates the transition of statistically significant differences in variance as sequence length increases. Based on pairwise Tukey HSD tests at each sequence length level, the ensemble classifiers result in significantly less variance at the 5 s sequence length than the group models (all p≤0.0069), while the opposite is true as sequence length increases to 30 s (all p≤0.0005). In the ensemble training methods, the strong trend of increasing inter-participant variance with increasing sequence length is again likely caused by the reduction in number of training sequences associated with an increase in sequence length. For ensemble models, this reduction is a higher proportion for each model than when all participants are grouped together. The result suggests to not only gather data from more participants, but also to gather more data for each individual if planning to use an ensemble method.

While the interaction of algorithm and training method was not significant, it should be noted that this was influenced by the removal of the 10 s sequence length LSTM point for the optimal-stopping validation-set group model whose accuracy was exceptionally low in comparison to all other models. Removal of this point could mask an interaction, but was required in order to satisfy ANOVA assumptions.

The statistical results do not tell the whole story regarding training technique. Digging a little deeper, we discovered that the validation-set approach was likely far more prone to overfitting than even these results indicate. Over a fifth (21.4%) of the training runs resulted in a maximum number of epochs condition being reached using the validation-set method. Post-hoc analysis of each of the cases that reached maximum epoch conditions revealed that 70.8% of the corresponding test accuracies were in severe overfitting conditions with performance rapidly decreasing compared to the perpetuating modest improvements of the remaining 29.2% of cases. While computational constraints placed an upper bound of 30 epochs on our training cases, had the validation-set approach been continued beyond the 30 epoch maximum, the performance gap between cross-validated and validation-set approaches undoubtedly would have widened since the cross-validated approach would have moderated these extremes through averaging. Additionally, using a cross-validated group-training technique resulted in improved accuracy compared to the optimal-stopping validation-set group training method for 26 out of 28 network architecture/sequence length combinations. While not examined in our analysis, future work could explore different methods for combining the number of training epochs from each fold during cross-validation, since taking the mean may not be the optimal method.

Finally, computational cost and experimental design considerations may guide a researcher to use a different technique than the cross-validated group method. Table 2 shows the computational cost difference between different cross-participant methods. The 7-classifier ensemble method was 35% more computationally efficient than the cross-validated group method and resulted in very similar performance to the first two training methods for the MPCRNN. Ensemble training methods are recommended when the set of individuals being modeled will change over time. If arrival of new users is expected, the 7-classifier ensemble (1 classifier per individual) is desirable because each model in the ensemble only requires data from a single individual for the training process, making integration of the new individual’s data into the existing ensemble an O(1) process. By comparison, the cross-validated group training method requires that with each new participant the entire model be retrained using data from all participants: adding an individual model to an existing model with *N* participants is O(N).

Due to the complex interactions associated with training method, for the remainder of our analysis we only consider the two recommended training methods: the cross-validated group method and the 7-classifier ensemble method. This greatly decreased the number of comparisons for the remaining factor analyses enabling a focused comparison of effects between these two recommended training methods.

### 3.2. Effect of Sequence Length

Figure 5 depicts the clear and statistically significant trend indicating that when holding other factors constant, increases in sequence length lead to improvements in model accuracy. This trend highlights the tradeoff between temporal specificity and predictive accuracy across all classifiers as sequence length increases. A longer sequence length means there is a decreased ability to identify and temporally locate workload transitions in real-time, which is a goal of our future research. In many applications, a reduction in classification accuracy associated with a reduced sequence length may be preferable to the loss of temporal specificity and the delay in predicting a change in the human’s cognitive workload.

The 3-factor ANOVA results modeling effects on mean accuracy are shown in Table 6. The associated model explained a large proportion of the variance with an $R^{2}=0.823$. Parameter estimates of the model indicated that sequence length was the most significant factor (p<0.0001) affecting mean classification accuracy with a 0.28% expected improvement for each additional second added to the sequence length.

Despite better predictive accuracy associated with longer sequence lengths, the variance among participants is not reduced. Based on a 3-factor cross-participant variance of classification accuracy ANOVA with first-order interaction terms for sequence length and training method (Table 7, $R^{2}=0.700$), all pertinent model parameters are positive, indicating that even after accounting for interaction effects, variance grows with increasing sequence length. This increase in variance occurs because the additional data present in longer sequence lengths allows participants whose models perform well at shorter sequence lengths to improve their performance, while simultaneously failing to enhance generalization performance on those participants whose models performed poorly at shorter sequence lengths. The distributional dissimilarity between participants is causing the divergence and indicates that increasing sequence length will not alleviate the problem of individual differences. Rather, we believe that an increased number of study participants and/or a novel set of features that can account for differences in cortical parcellation between participants, or which can capture participant-specific differences in task strategy, may be required to meaningfully improve cross-participant stationarity.

An important nuance regarding our treatment of sequence length compared to traditional treatments of increasing window length should be discussed. Previous results have indicated that longer windows for computing PSD tend to improve predictive performance [38,39,40]. However, these efforts used either average spectral power over increasingly long temporal periods, or merely increased the window length when computing PSD. This differs from our treatment of sequence length as a temporally ordered sequence of 2-s PSD computations with a 50% overlap, resulting in a 1 Hz update rate. This distinction is important because it means that there is potentially less of a tradeoff in temporal specificity compared to previous methods. However, since workload transitions were not present in our data, it is not possible to determine if improved temporal specificity is present even at longer sequence lengths. This is left for future work.

### 3.3. Effect of Model Architecture

The effect of model architecture was evaluated using the paired-down 3-factor ANOVAs (Table 6 and Table 7) and associated Tukey HSD tests when appropriate. We first present results of the cross-participant variance of classification accuracy ANOVA followed by the mean classification accuracy ANOVA.

Table 7 indicates that architecture had a significant effect on cross-participant variance of classification accuracy (p<0.0001). Significant results of an all-pairs Tukey HSD test comparing variance are shown in Table 8. The MPCRNN architecture produced models with less cross-participant variance than every other architecture (all p<0.0090) while all other architecture pairs had insignificant differences.

Table 6 showed architecture had a significant effect on mean classification accuracy (p=0.0006). Table 9 presents significant architecture pairs of a post-hoc Tukey HSD test. This analysis revealed that the MPCRNN statistically outperformed all other architectures in cross-participant model accuracy (all p<0.0443). Classification accuracy was improved on average by 2.2% to 3.2% absolute depending on the particular pair.

Due to the relationships between the MPCRNN, BDRLSTM, BDLSTM, and LSTM, analysis of these results suggest that there is a mean accuracy performance advantage associated with the addition of bidirectional context and residual connections to the 5-layer LSTM. However, the further addition of multi-path convolutional modules to the BDRLSTM resulted in an architecture which demonstrated superiority over all others. Notice that the only architecture which resulted in improved accuracy and decreased cross-participant variance compared to any other algorithm was the MPCRNN. This was significant because it means that the improvement in accuracy did not lead to the widening of cross-participant variance, as it did with sequence length. Rather, this architecture improved accuracy and led to a narrower difference between participants than all other tested architectures. This finding suggests that new feature representations can make headway on the challenge of building models that account for cross-participant distributional differences.

The MPCRNN performed better than all other architectures for both measures, indicating that these layers resulted in a learned representation which better matched assumptions about the data, and that those assumptions led to improved cross-participant generalization. The multi-path convolutional layers enabled the model to find global spatial correlates of brain activity and to learn useful cross-participant frequency representations (rather than using only features related to power in the standard clinical bands), while at the same time maintaining temporal ordering so that the recurrent network could account for temporal context. Additionally, the multi-path structure enabled a diversity of scale and representation that would otherwise not be possible. With each multi-path module, adjacent frequency bands could be pooled using maximum or average pooling, or adjacent bands could be convolved with filters that learn how to best combine information present at different frequencies. The 1×1 pass-through convolutional layers in each module also compressed the content of the current representation and allowed it to pass through to the next module enabling a diversity of scale not present in any other architecture. We posit that future use of multi-path architectures which enable not only frequential, but also localized spatial filtering, which, in combination with source localization techniques, may enable further improvements towards building a generalized architecture universally useful to multi-participant EEG analysis.

## 4. Conclusions

It is good to consider results in the context of outstanding challenges for a given field. In Section 1 we discussed the three main challenges associated with assessing operator functional state using psychophysiological features: temporal non-stationarity, individual differences, and cross-task applicability. Our work showed that using domain-specific knowledge to inform deep neural network architecture design resulted in a reduced impact on model performance due to individual differences. The cross-participant models produced a global reduction in cross-participant variance compared to within-participant models. Our results also showed that performance decrements due to within-participant temporal non-stationarity can be largely overcome by using models which incorporate other individuals’ data when sufficient diversity is present in the data.

For zero-data cross-participant modeling, we found that while increasing sequence length improves model accuracy, it does not improve generalizability since cross-participant variance increases due to cross-participant distributional differences. Furthermore, longer sequences reduce temporal specificity which decreases a model’s utility in a real-time environment. The only condition among our experiments across sequence lengths, architectures, and training methods which resulted in improved accuracy and decreased cross-participant variance was the multi-path convolutional recurrent architecture. The combination of multi-path convolutional layers with bidirectional context and residual connections enabled a diversity of scale and representation that better captured generalizable, cross-participant patterns of brain activity. Model training method was less important than other factors and should primarily be chosen based on experimental design and computational cost constraints; ensemble methods may be better if the underlying population being modeled will likely change frequently, but more data might be required per individual in order to offset the smaller amount of data available to train each model.

Our results so far suggest several avenues for future work to further improve cross-participant generalizability. Using models which learn more useful feature representations and gathering more data are the apparent paths for progress. A study employing a large number of participants (n>50) should be performed to understand how well deep networks can integrate and generalize psychophysiological data on a greater scale. It is expected that with greater diversity in the data set due to individual differences, the performance gap between within-participant and cross-participant modeling will further reduce—and with enough individuals available, group models may outperform individual models.

Unsupervised clustering of source localization across subjects using Independent Component Analysis (ICA) could also be attempted. This type of preprocessing may better align the input feature space with the assumptions of CNNs and also may improve recurrent network performance by pairing signaling from similar brain regions across subjects for each input feature. This would also enable a greater understanding of the underlying neuroscience at the expense of being able to perform real-time processing. Methods to weight ensemble models based on similarity of the input features to the feature distributions of each model in the ensemble could also be developed. This could allow for better cross-participant generalization by only using data from individuals with similar brain activity for a given task.

Future work is also required to improve cross-task applicability. Our results taken in context of other recent research which showed an improvement in temporal stationarity associated with the use of deep recurrent networks [30], demonstrates progress on two of the three primary assessment challenges in using deep neural networks—temporal non-stationarity of features, and cross-participant distributional differences. The progress made on these two challenges suggests that deep neural networks may be able to learn representations that have generalizable features at various depths—features that could be used to improve the third primary challenge, cross-task model applicability. We expect transfer learning using deep neural networks may provide a fundamentally different result than transfer learning using other techniques because deep networks compose low level features into hierarchical representations which may capture similarities across domains and allow for modifications of features from one domain to become relevant in the other.

## Figures and Tables

**Table 1:** Summary statistics for performance metrics and 2-factor ANOVA results evaluating if there is a difference in mean human performance between low and high workload by task. **Bold** denotes statistically significant results at the α=0.05 level of significance.

	Low Workload		High Workload		
Task	Mean	Sth Err	Mean	Sth Err	Prob > *F*
Tracking	95.2	4.1	64.3	7.8	**<0.0001**
System Monitoring	93.6	3.3	76.7	8.1	**<0.0001**
Communications	93.5	14.0	95.9	8.2	0.5783
Resource Mgmt	82.9	20.9	80.2	16.2	**<0.0001** *

* Significant difference between low and high workload for only participants 3 and 7.

**Table 2:** Mean classification accuracy across participants for a given training method, sequence length, and architecture. Number of parameters per model is displayed below each neural network architecture. Measured computational costs normalized to the cross-validated group model are displayed below each cross-participant training method. The best performing architecture for each training method and sequence length is shown in **bold**.

Training Method	Sequence Length	MPCRNN	BDRLSTM	BDLSTM	LSTM	2L-LSTM	CNN	ANN
{Normalized Computational Cost}		(6.2 M)	(4.2 M)	(4.2 M)	(1.9 M)	(0.7 M)	(1.8 M)	(1.1–6.5 M)
	5	**0.791**	0.768	0.760	0.766	0.771	0.635	0.749
Cross-Validated Group Model	10	**0.820**	0.785	0.777	0.778	0.793	0.657	0.777
{1.0}	20	**0.850**	0.839	0.839	0.811	0.820	0.732	0.834
	30	**0.868**	0.852	0.853	0.849	0.834	0.711	0.862
	5	**0.781**	0.748	0.748	0.682	0.754	0.627	0.743
Optimal-Stopping Val-Set Group Model	10	**0.819**	0.786	0.785	0.689	0.782	0.599	0.774
{0.12}	20	**0.834**	0.825	0.825	0.802	0.809	0.684	0.828
	30	0.850	**0.860**	0.846	0.838	0.824	0.691	0.855
	5	**0.791**	0.775	0.771	0.757	0.773	0.731	0.767
7-Classifier Ensemble Model	10	**0.822**	0.806	0.805	0.789	0.797	0.757	0.798
{0.65}	20	**0.842**	0.814	0.803	0.833	0.798	0.757	0.834
	30	**0.865**	0.833	0.812	0.808	0.808	0.732	0.838
	5	0.768	**0.780**	0.778	0.765	0.773	0.701	0.769
28-Classifier Ensemble Model	10	0.800	0.804	**0.806**	0.792	0.801	0.690	0.802
{0.52}	20	0.809	0.807	0.816	0.826	0.799	0.731	**0.833**
	30	0.837	0.825	0.818	0.809	0.810	0.694	**0.841**

**Table 3:** Results of the 3-factor ANOVA evaluating the effects of architecture, sequence length, training method, and first-order interactions on mean classification accuracy. Significant results are displayed in **bold**.

Factor	DF	Sum of Squares	*F* Ratio	Prob > *F*
Architecture	5	0.0090	10.0929	**<0.0001**
Sequence Length	1	0.0709	398.2276	**<0.0001**
Training Method	3	0.0021	3.9591	**0.012**
Sequence Length*Training Method	3	0.0073	13.7292	**<0.0001**
Architecture*Sequence Length	5	0.0029	3.2372	**0.0116**
Architecture*Training Method	15	0.0039	1.4701	0.1449

**Table 4:** Tukey HSD pairwise comparisons for the interaction of training method and sequence length at specified sequence lengths. Significant results are shown in **bold**.

		5 s				10 s				20 s				30 s			
Comparison		Est	Sth Err	t Ratio	Pr >|t|	Est	Sth Err	t Ratio	Pr >|t|	Est	Sth Err	t Ratio	Pr >|t|	Est	Sth Err	t Ratio	Pr >|t|
28-Class	7-Class	−0.001	0.006	−0.192	0.9975	−0.002	0.005	−0.421	0.9746	−0.004	0.004	−0.854	0.8284	−0.005	0.007	−0.762	0.8713
28-Class	CV Grp	0.011	0.006	1.800	0.2832	0.003	0.005	0.565	0.9421	−0.014	0.004	−3.282	**0.0090**	−0.030	0.007	−4.420	**0.0002**
28-Class	Val Set	0.030	0.006	4.886	**<0.0001**	0.019	0.005	4.034	**0.0009**	−0.002	0.004	−0.555	0.9448	−0.024	0.007	−3.500	**0.0047**
7-Class	CV Grp	0.012	0.006	1.992	0.2022	0.005	0.005	0.986	0.7579	-0.010	0.004	−2.428	0.0823	−0.025	0.007	−3.658	**0.0029**
7-Class	Val Set	0.031	0.006	5.074	**<0.0001**	0.021	0.005	4.447	**0.0002**	0.001	0.004	0.293	0.9912	−0.018	0.007	−2.738	**0.0392**
CV Grp	Val Set	0.019	0.006	3.120	**0.0142**	0.016	0.005	3.481	**0.0050**	0.011	0.004	2.704	**0.0427**	0.006	0.007	0.920	0.7941

**Table 5:** Results of the 3-factor ANOVA evaluating the effects of architecture, sequence length, training method, and first-order interactions on cross-participant variance of classification accuracy. Significant results are displayed in bold.

Source	DF	Sum of Squares	*F* Ratio	Prob > *F*
Architecture	5	0.00033601	16.3957	**<0.0001**
Sequence Length	1	0.00042976	104.8501	**<0.0001**
Training Method	3	0.00001822	1.4816	0.2283
Sequence Length*Training Method	3	0.00037315	30.3464	**<0.0001**
Architecture*Sequence Length	5	0.00011529	5.6258	**0.0002**
Architecture*Training Method	15	0.00010311	1.6771	0.0796

**Table 6:** Results of the 3-factor ANOVA evaluating the main effects of architecture, sequence length, and two training methods (cross-validated group method and 7-classifier ensemble method) on mean accuracy. Significant results are displayed in **bold**.

Factor	DF	Sum of Squares	*F* Ratio	Prob > *F*
Architecture	5	0.0058	5.4780	**0.0006**
Sequence Length	1	0.0335	157.0921	**<0.0001**
Training Method	1	0.0003	1.1776	0.2843

**Table 7:** Results of the 3-factor ANOVA evaluating the main effects of architecture, sequence length, and two training methods (cross-validated group method and 7-classifier ensemble method), including the interaction effect of training method and sequence length, on cross-participant variance of classification accuracy. Significant results are displayed in **bold**.

Factor	DF	Sum of Squares	*F* Ratio	Prob > *F*
Architecture	5	0.0002	7.2180	**<0.0001**
Sequence Length	1	0.0002	38.5229	**<0.0001**
Training Method	1	0.0000	0.1122	0.7395
Sequence Length*Training Method	1	0.0001	15.6379	**0.0003**

**Table 8:** Post-hoc Tukey HSD results between architectures, for the cross-participant variance of classification accuracy ANOVA. Only significant results are shown (α = 0.05).

Arch 1	Arch 2	Diff	Std Err	*t* Ratio	Prob > |t|	95% Conf Int
MPCRNN	BDLSTM	−0.0062	0.0012	5.02	0.0002	−0.0100 to −0.0025
MPCRNN	2L-LSTM	−0.0061	0.0012	4.87	0.0003	−0.0098 to −0.0023
MPCRNN	BDRLSTM	−0.0058	0.0012	4.69	0.0004	−0.0096 to −0.0021
MPCRNN	LSTM	−0.0052	0.0012	4.21	0.0019	−0.0090 to −0.0015
MPCRNN	ANN	−0.0046	0.0012	3.66	0.0090	−0.0083 to −0.0008

**Table 9:** Post-hoc Tukey HSD results between architectures, for the mean classification accuracy ANOVA. Only significant results are shown (α = 0.05).

Arch 1	Arch 2	Diff	Std Err	*t* Ratio	Prob > |t|	95% Conf Int
MPCRNN	LSTM	0.032	0.0073	4.44	0.0009	0.0105 to 0.0542
MPCRNN	2L-LSTM	0.032	0.0073	4.37	0.0011	0.0100 to 0.0538
MPCRNN	BDLSTM	0.029	0.0073	3.93	0.0041	0.0068 to 0.0506
MPCRNN	ANN	0.024	0.0073	3.30	0.0233	0.0022 to 0.0469
MPCRNN	BDRLSTM	0.022	0.0073	3.04	0.0443	0.0004 to 0.0441

## Supplementary Materials

The following are available online at http://www.mdpi.com/1424-8220/18/5/1339/s1.
