# Supplementary material for: Cross-Participant EEG-Based Assessment of Cognitive Workload Using Multi-Path Convolutional Recurrent Neural Networks
Source: Sensors (Basel). 2018 Apr 26;18(5):1339. doi: 10.3390/s18051339 (PMC5982227; doi:10.3390/s18051339)
Supplement: Supplementary file 1 [file sensors-18-01339-s001.zip › Supplementary Materials/2L-LSTM.pdf]

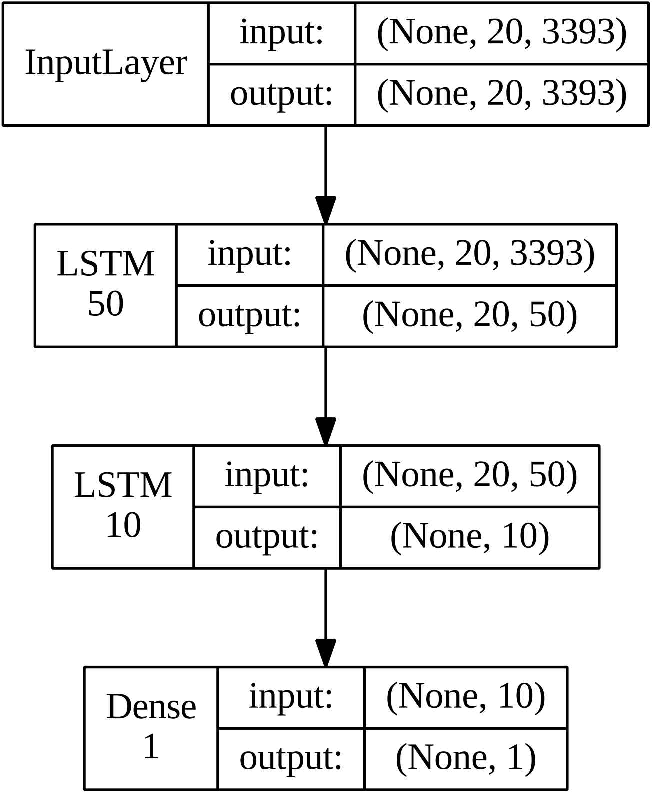
InputLayer
input:
(None, 20, 3393)
output:
(None, 20, 3393)
LSTM
50
input:
(None, 20, 3393)
output:
(None, 20, 50)
LSTM
10
input:
(None, 20, 50)
output:
(None, 10)
Dense
1
input:
(None, 10)
output:
(None, 1)
