# Supplementary material for: Cross-Participant EEG-Based Assessment of Cognitive Workload Using Multi-Path Convolutional Recurrent Neural Networks
Source: Sensors (Basel). 2018 Apr 26;18(5):1339. doi: 10.3390/s18051339 (PMC5982227; doi:10.3390/s18051339)
Supplement: Supplementary file 1 [file sensors-18-01339-s001.zip › Supplementary Materials/BDRLSTM.pdf]

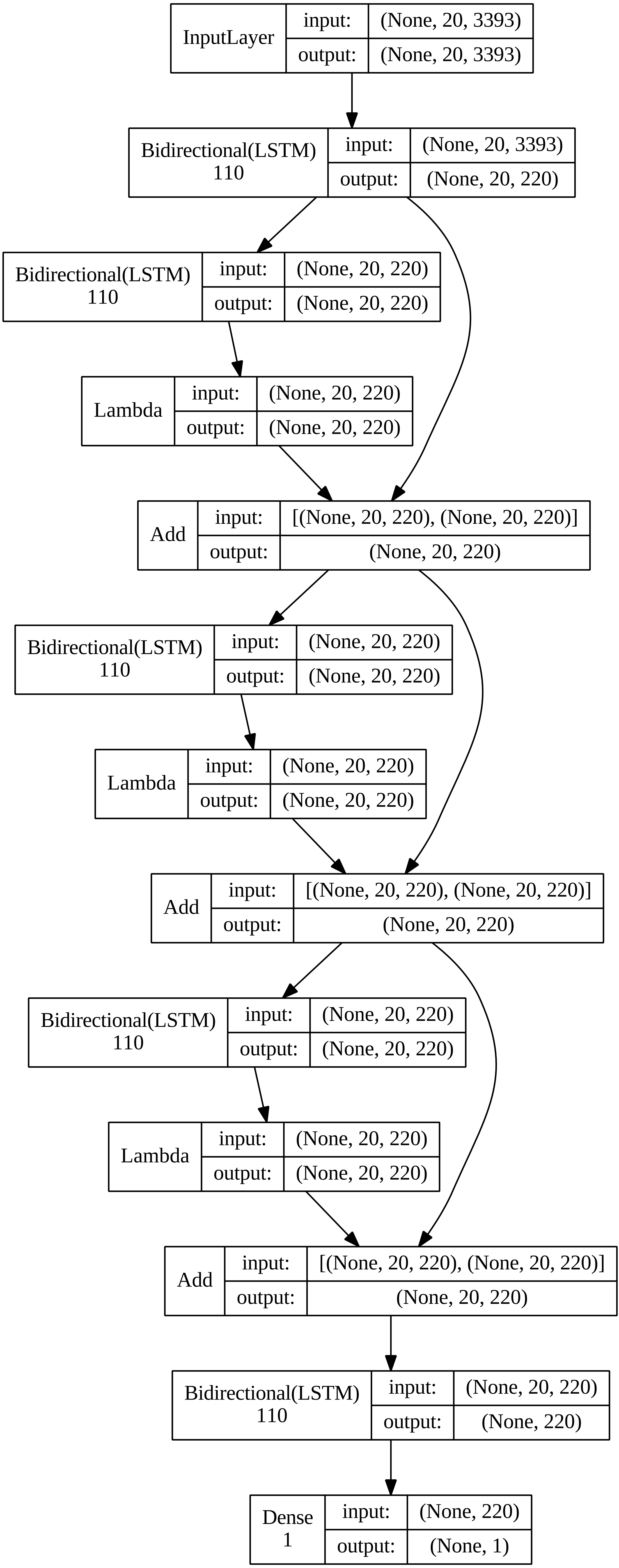

InputLayer
input: (None, 20, 3393)
output: (None, 20, 3393)
Bidirectional(LSTM) 110
input: (None, 20, 3393)
output: (None, 20, 220)
Bidirectional(LSTM) 110
input: (None, 20, 220)
output: (None, 20, 220)
Lambda
input: (None, 20, 220)
output: (None, 20, 220)
Add
input: [(None, 20, 220), (None, 20, 220)]
output: (None, 20, 220)
Bidirectional(LSTM) 110
input: (None, 20, 220)
output: (None, 20, 220)
Lambda
input: (None, 20, 220)
output: (None, 20, 220)
Add
input: [(None, 20, 220), (None, 20, 220)]
output: (None, 20, 220)
Bidirectional(LSTM) 110
input: (None, 20, 220)
output: (None, 20, 220)
Lambda
input: (None, 20, 220)
output: (None, 20, 220)
Add
input: [(None, 20, 220), (None, 20, 220)]
output: (None, 20, 220)
Bidirectional(LSTM) 110
input: (None, 20, 220)
output: (None, 220)
Dense 1
input: (None, 220)
output: (None, 1)
