# Supplementary material for: Cross-Participant EEG-Based Assessment of Cognitive Workload Using Multi-Path Convolutional Recurrent Neural Networks
Source: Sensors (Basel). 2018 Apr 26;18(5):1339. doi: 10.3390/s18051339 (PMC5982227; doi:10.3390/s18051339)
Supplement: Supplementary file 1 [file sensors-18-01339-s001.zip › Supplementary Materials/CNN.pdf]

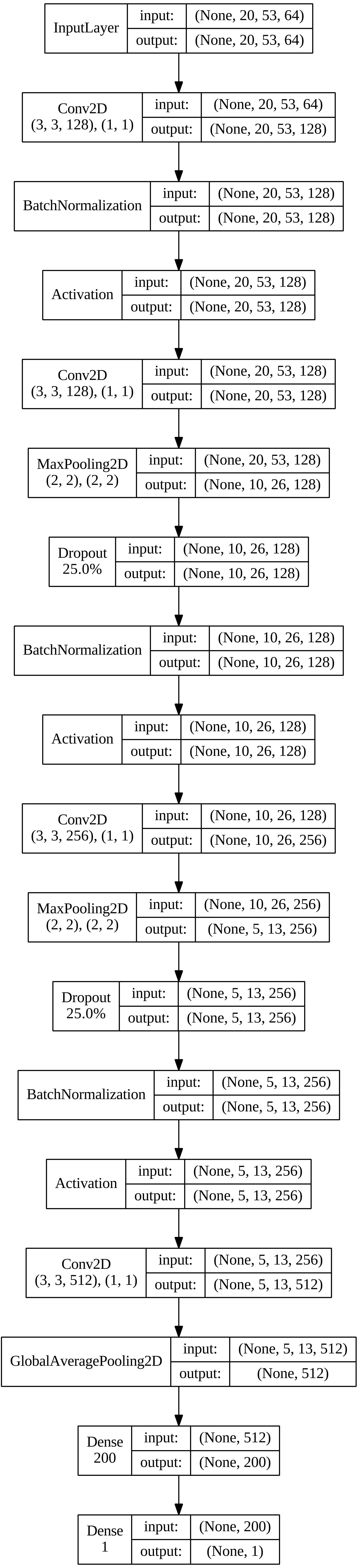

InputLayer
input: (None, 20, 53, 64)
output: (None, 20, 53, 64)
Conv2D
(3, 3, 128), (1, 1)
input: (None, 20, 53, 64)
output: (None, 20, 53, 128)
BatchNormalization
input: (None, 20, 53, 128)
output: (None, 20, 53, 128)
Activation
input: (None, 20, 53, 128)
output: (None, 20, 53, 128)
Conv2D
(3, 3, 128), (1, 1)
input: (None, 20, 53, 128)
output: (None, 20, 53, 128)
MaxPooling2D
(2, 2), (2, 2)
input: (None, 20, 53, 128)
output: (None, 10, 26, 128)
Dropout
25.0%
input: (None, 10, 26, 128)
output: (None, 10, 26, 128)
BatchNormalization
input: (None, 10, 26, 128)
output: (None, 10, 26, 128)
Activation
input: (None, 10, 26, 128)
output: (None, 10, 26, 128)
Conv2D
(3, 3, 256), (1, 1)
input: (None, 10, 26, 128)
output: (None, 10, 26, 256)
MaxPooling2D
(2, 2), (2, 2)
input: (None, 10, 26, 256)
output: (None, 5, 13, 256)
Dropout
25.0%
input: (None, 5, 13, 256)
output: (None, 5, 13, 256)
BatchNormalization
input: (None, 5, 13, 256)
output: (None, 5, 13, 256)
Activation
input: (None, 5, 13, 256)
output: (None, 5, 13, 256)
Conv2D
(3, 3, 512), (1, 1)
input: (None, 5, 13, 256)
output: (None, 5, 13, 512)
GlobalAveragePooling2D
input: (None, 5, 13, 512)
output: (None, 512)
Dense
200
input: (None, 512)
output: (None, 200)
Dense
1
input: (None, 200)
output: (None, 1)
