# Supplementary figures and images for: Cross-Participant EEG-Based Assessment of Cognitive Workload Using Multi-Path Convolutional Recurrent Neural Networks
Source: Sensors (Basel). 2018 Apr 26;18(5):1339. doi: 10.3390/s18051339 (PMC5982227; doi:10.3390/s18051339)

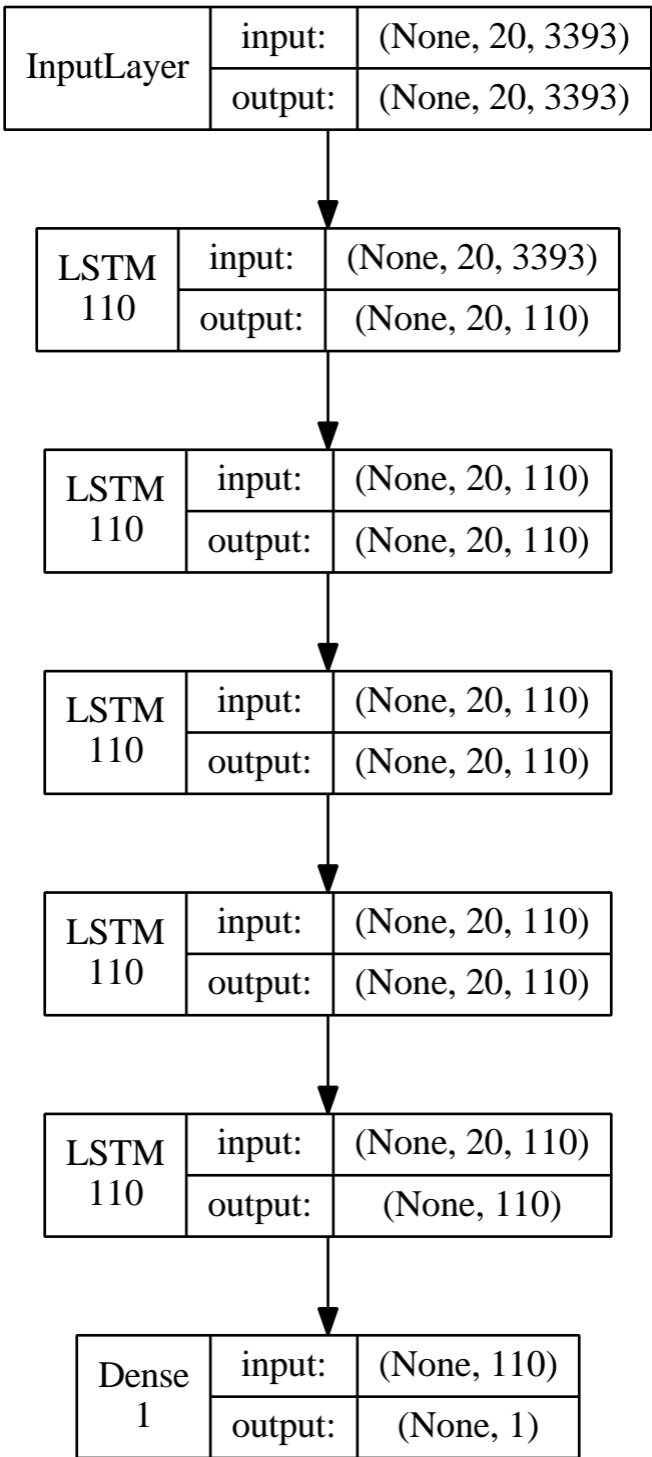

Supplement: Supplementary file 1 [file sensors-18-01339-s001.zip › Supplementary Materials/LSTM.pdf]

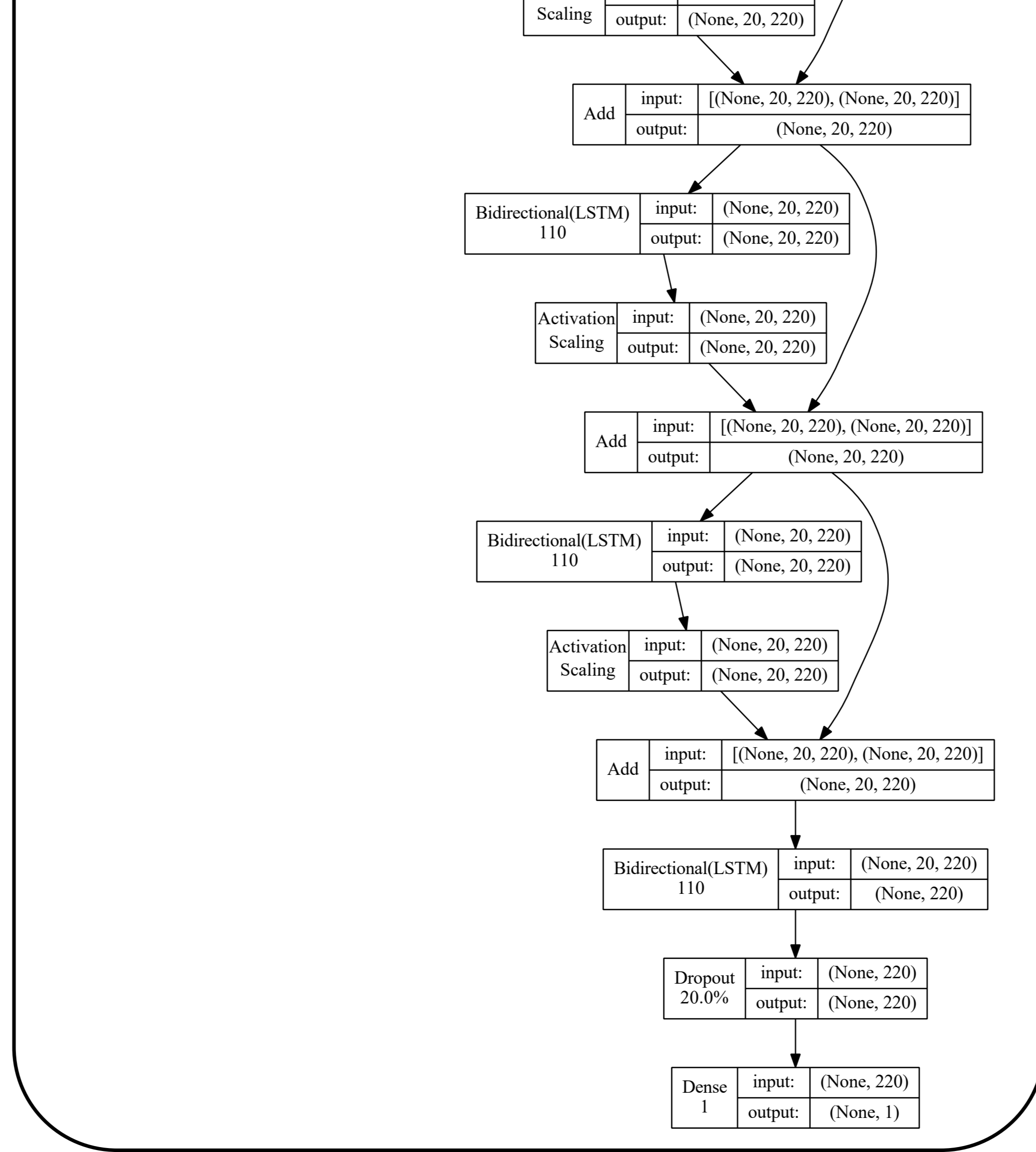

Supplement: Supplementary file 1 [file sensors-18-01339-s001.zip › Supplementary Materials/MPCRNN3.pdf]
